# Medication Abortion Failure in a Patient With Class III Obesity and Twin Gestation: A Case Report

**DOI:** 10.7759/cureus.107285

**Published:** 2026-04-18

**Authors:** Dan Schaefer, Aaron Brown, Melissa A Adler, J. Bradley Stern

**Affiliations:** 1 Department of Family Medicine, University of Colorado Health, Fort Collins, USA; 2 Department of Pharmacology, University of Wyoming, Laramie, USA; 3 Department of Obstetrics and Gynecology, University of Colorado School of Medicine, Aurora, USA

**Keywords:** abortion failure, class iii obesity, clinical pharmacokinetics, extreme obesity, medication abortion, twin gestation, women's health

## Abstract

There is a paucity of clinical data stratifying medication abortion (MAB) success rates by obesity class or multifetal gestation. With rising rates of class III obesity, it is important to assess the efficacy of current MAB protocols in this population. A 30-year-old G3P1011 at six weeks two days (6w2d) with a diamniotic, dichorionic twin gestation and a body mass index (BMI) of 53, elected for MAB. She received 200 mg oral mifepristone followed by two doses of 800 mcg buccal misoprostol over 48 hours. Minimal symptoms occurred initially, and she ultimately required manual vacuum aspiration 31 days later for retained products. This case highlights possible limitations of standard MAB regimens in class III obesity with twin gestation, suggesting altered pharmacokinetics and the need for further research to optimize care.

## Introduction

Medication abortion (MAB) is an important component of reproductive healthcare that is currently available in 36 U.S. states. The American College of Obstetricians and Gynecologists (ACOG) has previously released a practice bulletin on MAB up to 70 days of gestation that provides guidance on how to perform medication abortion [1]. ACOG also has a formal committee opinion on increasing access to abortion, including utilizing MAB when able [2]. Although medication abortion using mifepristone and misoprostol is highly effective, failure rates vary, and predictors of failure remain poorly defined, particularly in special populations.

Obesity affects over 11% of U.S. adult women [3], yet there are no current studies on MAB that stratify by obesity class [4,5]. Additionally, limited data exist on the efficacy of MAB in twin gestations. Although literature suggests that medication abortion success rates are similar between singleton and twin pregnancies up to 63 days of gestation [6,7], it does not address the impact of obesity on these success rates. Alternatively, pharmacokinetic concerns have been raised due to the lipophilic nature of mifepristone and misoprostol, and the influence of administration route [8-14], but no studies have explored whether obesity and twin gestation, including specific subtypes such as dichorionic diamniotic pregnancies, together affect outcomes.

We report a case of medication abortion failure in a patient with class III obesity and dichorionic, diamniotic twin gestation despite receiving the standard combination regimen with a second misoprostol dose.

## Case presentation

The patient was a 30-year-old woman, G3P1011, at six weeks two days (6w2d) by formal ultrasound, with an uncertain last menstrual period approximately seven weeks prior. A formal ultrasound at five weeks five days (5w5d) had shown an intrauterine pregnancy consisting of diamniotic, dichorionic twins (Video 1). Her past medical history was notable for depression, anxiety, mild persistent asthma, juvenile myoclonic epilepsy, hypertension, and class III obesity with a body mass index (BMI) of 53 on intake. The patient presented to the clinic seeking an abortion. She had undergone a manual vacuum aspiration (MVA) in the past and described it as a traumatic experience, electing for a medication abortion after a risk and benefits discussion. Her vital signs were within normal limits, and the physical examination was unremarkable. Current medications included budesonide, cholecalciferol, folic acid, labetalol, levetiracetam, norethindrone acetate-ethinyl estradiol, and prenatal vitamins. As-needed medications included acetaminophen, albuterol, and alprazolam.

**Video 1:** Transvaginal ultrasound of early diamniotic, dichorionic twin pregnancy. Transvaginal ultrasound performed at five weeks five days gestation demonstrating a viable diamniotic, dichorionic twin intrauterine pregnancy.

The patient consented to MAB after review of her medical and surgical history, which revealed no contraindications or special considerations. The administration of mifepristone 200 mg orally was observed by the ordering physician in the clinic. The patient was then dispensed 800 mcg of misoprostol and given instructions for buccal administration 24 hours later. Approximately 30 hours after the initial mifepristone administration, the patient called the clinic due to a lack of cramping and bleeding following medication administration. She was informed that it might take up to 48 hours to feel the effects and was scheduled for an appointment the following day to coincide with the 48-hour mark. At approximately 48 hours after the initial mifepristone dose and 24 hours after the initial misoprostol dose, a second dose of 800 mcg misoprostol was administered buccally in the clinic. Approximately 72 hours after the initial mifepristone administration, the patient began experiencing bleeding and cramping and reported passing clotted blood.

The patient was then scheduled for an ultrasound 29 days after the initial dose to evaluate for retained products. Endovaginal ultrasound at that time demonstrated heterogeneous tissue within the superior aspect of the uterus measuring 19 mm with increased peripheral vascularity, concerning for retained products of conception. The patient subsequently underwent an uncomplicated MVA, 31 days after the initial dose of mifepristone.

Patient-reported outcomes

The patient reflected on her care experience, noting: "My experience with medical and surgical abortion as an obese woman has been very challenging-physically, mentally, and emotionally. No one prepares you for the amount of guilt, shame, and disgust you feel during and after this process. As someone who has been obese their whole life, I never once had an issue with my size as it related to my reproductive health. But, that all changed during this process. While the majority of my care team met me with kindness, understanding, and compassion for my situation (I have a nonverbal autistic toddler and another pregnancy-nonetheless, twins, just didn't align with the cards I have been dealt as a support parent). I've been on the pill since I was roughly 12 years old. I didn't take my medication correctly in college, which is how I first learned the horrors of a medical abortion at 20 years old. I swore I would never do that again. But here I was, 30 years old, and in the same boat. I hadn't been taking my medication correctly. During this process, I had a doctor tell me that “because of my size, the pill probably won't work as effectively,” not “you need to be on a different form of birth control if you are going to keep forgetting to take it as directed, idiot”. I will never forget those words and I will never not tell someone why I currently have an IUD- because I am “too fat” for the pill. Am I also too fat for the abortion pill? Seems so. After two filled rounds of the abortion pill, I had no choice but to open old emotional wounds and have another surgical abortion. It was sickening, traumatizing, and soul-crushing. I encourage any doctors interested in this topic to research and then research more so that the next woman has a more successful outcome. I'm thankful to be a part of this journal so that my story and experience can end up in the right hands, and help other women like me safe and healthy abortion journey-regardless of weight. A special thank you to my doctor, an incredible doctor, and even more incredible human-your compassion and care will never be forgotten by me."

## Discussion

This case illustrates medication abortion failure in a patient with class III obesity (BMI 53) and a diamniotic, dichorionic twin pregnancy, despite the appropriate use of mifepristone and two doses of buccal misoprostol. The patient ultimately required surgical management via MVA.

Studies on early pregnancy loss demonstrate that the combination of mifepristone and misoprostol is more effective than misoprostol alone, with efficacy further improved by a second dose of misoprostol [15]. While research has explored predictors for the need for evacuation after MAB and the effects of obesity on medical abortion, no studies have stratified success rates by obesity class [4,5]. Class III obesity is defined as a BMI of 40 or more, and neither mifepristone nor misoprostol has specific weight-based dosing guidelines.

There is limited pharmacokinetic data to guide dosing of mifepristone and misoprostol for medication abortion in patients with class III obesity. Mifepristone, a highly lipophilic drug, may have an increased volume of distribution (Vd) and clearance in obese individuals, potentially leading to lower plasma concentrations and reduced efficacy [8-11]. Misoprostol, also lipophilic, is typically administered buccally, though vaginal administration has been shown to result in higher systemic drug exposure [12-14]. However, there is a proposed synergistic effect between mifepristone and misoprostol, and no significant difference in uterine contractility between routes of misoprostol administration [16]. Given that obese patients may experience increased drug clearance and distribution, buccal misoprostol may lead to suboptimal serum levels. Research on obese patients undergoing first-trimester abortion suggests they may require additional doses of misoprostol and experience longer times to uterine evacuation, raising concerns about reduced MAB effectiveness in this population [17]. Higher doses or alternative administration routes, such as vaginal misoprostol, may be needed to optimize success. No clinically significant drug-drug interactions were identified between mifepristone or misoprostol and the patient’s home medications that would be expected to impact treatment efficacy.

Current studies evaluating the effects of obesity on MAB have not demonstrated statistically significant differences in rates of surgical intervention but do not stratify by obesity class, instead categorizing patients dichotomously as BMI less than or greater than 30 [4]. Other studies have identified parity of five or more as a predictor of MVA following medication abortion [18], which does not apply to this patient.

In addition to obesity, there is a paucity of data regarding MAB outcomes across different types of twin gestations. Existing studies evaluating medication abortion in twin pregnancies do not stratify outcomes by chorionicity or amnionicity, and no data are available comparing failure rates among specific subtypes such as monochorionic monoamniotic, monochorionic diamniotic, or dichorionic diamniotic gestations. Furthermore, no studies have evaluated the interaction between obesity class and twin gestation subtype on MAB success. Differences in placental mass, hormone levels, and uterine distension across twin subtypes may theoretically influence response to medication abortion, though this has not been studied. Accordingly, it remains unclear whether specific twin subtypes confer differential risk of medication abortion failure. Given the available evidence, it is not possible to determine whether treatment failure in this case is primarily attributable to class III obesity, twin gestation, or the combined effect of both factors.

## Conclusions

This case of medication abortion failure in a patient with class III obesity treated with oral mifepristone and two doses of buccal misoprostol adds to the current literature. It suggests the need for further studies that consider both twin gestation and obesity class stratification when examining MAB success rates. With a significant portion of adult women in the U.S. currently living with a BMI placing them in the class III obesity category, additional clinical studies should be conducted on the safety and efficacy of MAB in this significant population. These gaps in the existing literature underscore the need for further research that stratifies outcomes by obesity class, evaluates weight-based dosing strategies, and examines the impact of multiple gestations on MAB efficacy, both independently and in combination. This is necessary to ensure safe and effective access to MAB for all patient populations, and to support accurate, individualized risk-benefit discussions.
